# Motivational component profiles in university students learning histology: a comparative study between genders and different health science curricula

**DOI:** 10.1186/1472-6920-14-46

**Published:** 2014-03-10

**Authors:** Antonio Campos-Sánchez, Juan Antonio López-Núñez, Víctor Carriel, Miguel-Ángel Martín-Piedra, Tomás Sola, Miguel Alaminos

**Affiliations:** 1 Department of Histology, School of Medicine, University of Granada, Avenida de Madrid, 11 E18071 Granada, Spain; 2 Department of Didactics and School Organization, School of Education, University of Granada, Campus de Cartuja, E18071 Granada, Spain

**Keywords:** Motivation, Components, Curriculum, Histology, Health sciences

## Abstract

**Background:**

The students’ motivation to learn basic sciences in health science curricula is poorly understood. The purpose of this study was to investigate the influence of different components of motivation (intrinsic motivation, self-determination, self-efficacy and extrinsic -career and grade- motivation) on learning human histology in health science curricula and their relationship with the final performance of the students in histology.

**Methods:**

Glynn Science Motivation Questionnaire II was used to compare students’ motivation components to learn histology in 367 first-year male and female undergraduate students enrolled in medical, dentistry and pharmacy degree programs.

**Results:**

For intrinsic motivation, career motivation and self-efficacy, the highest values corresponded to medical students, whereas dentistry students showed the highest values for self-determination and grade motivation. Genders differences were found for career motivation in medicine, self-efficacy in dentistry, and intrinsic motivation, self-determination and grade motivation in pharmacy. Career motivation and self-efficacy components correlated with final performance in histology of the students corresponding to the three curricula.

**Conclusions:**

Our results show that the overall motivational profile for learning histology differs among medical, dentistry and pharmacy students. This finding is potentially useful to foster their learning process, because if they are metacognitively aware of their motivation they will be better equipped to self-regulate their science-learning behavior in histology. This information could be useful for instructors and education policy makers to enhance curricula not only on the cognitive component of learning but also to integrate students’ levels and types of motivation into the processes of planning, delivery and evaluation of medical education.

## Background

The affective or motivational dimension of the educational process is important because of its influence on students’ learning behavior. Motivation affects the not only the decision to begin, persevere in or end a specific learning behavior, but also the choice of a specific behavior [1]. Accordingly, motivation constitutes a key element in education research, and many theories have been put forward to account for the nature and influence of motivation in the learning process [2-4]. Some of the most relevant theories of motivation [5] are the theory of the hierarchy of needs [6], the theory of the need to achieve [7], the expectancy value theory [8], the attribution theory [9], the self-determination theory [10], the goal theory [11], and the social cognitive theory [12,13]. According to Social Cognitive Theory [12,14], motivation is understood as a series of reciprocal interactions among environmental contexts, behaviors and personal characteristics [15]. This theory views students’ learning as most effective when, according to Glynn and cols. [16], it is self-regulated, which occurs when students assume conscious control over their motivation and behavior in a way which leads to desirable learning outcomes. Within this theoretical framework, the motivation to learn science is defined as an internal state that maintains science-learning behavior, and as a multicomponent construct made up of, among other components, intrinsic motivation, extrinsic motivation, self-determination and self-efficacy [3,16], which are supported by different theories [10,17-19]. According to several authors [16,20], intrinsic motivation involves the inherent satisfaction produced by acquiring new science knowledge; extrinsic motivation involves learning science as a means to a concrete end [21]; self-determination refers to the control that students think they have over their science learning [22]; and self-efficacy refers to the students’ conviction that they can achieve well in science [23,24]. One of the innovative aspects recently introduced by Glynn and cols. [16] is the transformation of the classical scale termed extrinsic motivation into two scales, i.e. grade motivation, related to short-term goals, and career motivation, related to long-term goals, which more clearly target the objectives that students perceive to be important in this stage of their education [25].

Several authors [16,26-28] have provided substantial contributions to research on the motivations to learn science, and have yielded validated questionnaires to assess these motivations with a set of observable variables (items) that serve as empirical indicators. These authors have investigated some of these components in different science disciplines and reported that they influence behavior and self-regulation in students’ learning process [21,22,24].

Motivation is recognized as an important factor in health science education because it helps students achieve good academic performance, well-being and satisfaction, and also helps them to become good professionals. However, research that centers on motivation in the health science disciplines is scarce [5,17,29]. This factor is especially important in learning basic sciences such as gross anatomy, histology and physiology in health science curricula. Although research on students’ motivation to learn science has increased during the last two decades, little attention has focused on motivation to learn basic sciences that are considered essential requirements in health science curricula [26,30-32].

Human histology is a branch of Biology and Health Sciences dealing with the study of levels of organization that are intercalated between the atomic-molecular level (Biochemistry) and the gross morphological level (Anatomy) in the human body. Medical, Dentistry and Pharmacy programs include histology as part of their basic or preclinical curricula [33-35]. Although histology has traditionally been taught as a lecture- and microscopy-based course, new approaches have recently been used to emphasize self-learning processes such as virtual microscopy, team-based peer teaching and learning, and clinical-histologic conferences [36-38]. Nonetheless, histology is perceived by students as an abstract subject which is generally difficult to understand, and many students find it challenging to connect theory with practice [39]. Furthermore, health sciences students often fail to appreciate the relevance of learning and understanding the normal structures and functions of the body for their future clinical or professional activities [34,40].

Research into the motivations to learn science has thus far paid little attention to basic sciences in the health science curricula [41]. Motivation is especially important in human histology not only because of the conceptual difficulty of the subject, but also because of the difficulty of projecting the affective factors associated with its applications to medicine and other health sciences to the learning process [42]. As a result, the influence of different components of motivation (e.g. intrinsic motivation, self-determination, self-efficacy and extrinsic motivation) on human histology learning in health science curricula is poorly understood.

As noted by Brian and cols. [15], the different components of motivation have been studied in science education, but usually alone rather than in relation to each other. A question we want to investigate in the present study was the interrelation among different components in students of human histology enrolled in three different undergraduate health science programs, and to determine if the components of motivation are related to histology course performance. Because different components of motivation can influence students’ science-learning behavior, knowledge about the influence of each component will be useful to more effectively foster students’ motivation to learn histology in health sciences [15,43]. Furthermore, some previous studies focused on the influence of gender on motivation and achievement in medical schools [44,45]. However, studies carried out by Kusurkar and cols. do not really connect strength of motivation to performance in medical schools [46]. Yet, the incidence of gender on the different components of motivation of health sciences students has been poorly studied. For these reasons, another question of the present study was to investigate male and female students’ motivations to learn human histology in medical, dentistry and pharmacy curricula and to determine the incidence of gender on the participation of the different components of motivation.

## Methods

### Design of the study

This study was carried out to evaluate the motivational component profiles in university students learning histology using a validated questionnaire described by Glynn and cols [16]. To compare the results among different health science curricula, the study was carried out during the same course, in the same period of the year and with instructors belonging to the same Department -Department of Histology-, which is responsible for teaching histology in Medicine, Dentistry and Pharmacy Schools. This study was approved by the Ethics and Research Committee of the University of Granada.

### Sample

The study was done at the School of Medicine, School of Dentistry and School of Pharmacy at the University of Granada in Granada, Spain. The sample consisted of 367 first-year undergraduate students (average age 18 years) enrolled in the histology course that formed part of their medical, dentistry or pharmacy curriculum in accordance with European Union regulations. In all these degrees, Histology is a basic science subject included in the core curriculum. Teaching is carried out by using lectures, practical sessions with the use of microscopes, and sessions with histology problem-solving strategies. There were 132 participants (43 males and 89 females) at the medical school, 125 (44 males and 81 females) at the dentistry school and 110 (31 males and 79 females) at the pharmacy school. All participants agreed to participate in the study. The students’ participation was voluntary and consistent with the procedures of the university research review boards. The students were given no extra credit or compensation for participating. They were informed that their participation would help improve histology instruction. The final performance of the students in the histology subject was recorded at the end of the course to be used in the study.

### Instrument

To evaluate the different components of the motivation to learn histology in students at the medical, dentistry and pharmacy schools, all participants responded to the Science Motivation Questionnaire II (SMQII) developed by Glynnn and cols. [16], which uses five items to assess each of the five components of motivation: intrinsic motivation, self-determination, self-efficacy, career motivation and grade motivation. A total of 25 items were evaluated in each student. The items in the SMQII were designed to serve as empirical indicators of components of students’ motivation not only to learn science in university courses but also to learn a specific science discipline. The instrument is readily adapted to specific disciplines by replacing the word “science” in each item with the name of the discipline of interest, as noted by Glynn and cols. [16]. In the present study we used the word “histology” instead of “science” to focus on this specific course, which is taught as part of three different undergraduate health science degree programs at our university, and we have used Spanish language -native for the students- instead of English. We previously used basic criteria reported previously [47] to verify that each item was representative for the target discipline, which is taught by instructors from the same department at the University of Granada. As recommended by DeVellis [48], the items were randomly sorted, strongly worded, unambiguous declarative statements in the form of short, simple sentences without jargon. For these reasons, the order of the items -originally sorted by the authors- was preserved in our Spanish translation of the questionnaire. Students rated each item on a five-point type scale: never, rarely, sometimes, often or always. The questionnaire was administered during classroom instruction in an advanced period of the course. The students were first briefed on the purpose of the instrument and given instructions about how to complete the questionnaire, which took about 15 min to complete. The questionnaire was used with permission from the SMQII website hosted by the University of Georgia at http://www.coe.uga.edu/smq/.

### Statistical analysis

Average values and standard deviations were calculated for each item, for each group of students and for each gender. Mean values were also calculated for each component of motivation by determining the average value of the 5 items included in each component. To compare the results between males and female students or between two different curricula (medicine vs. dentistry, medicine vs. pharmacy and dentistry vs. pharmacy) we used the Mann-Whitney test, since the variables did not fit the normal distribution as demonstrated by the Kolmogorov-Smirnov test. Finally, to determine if the final performance of the students in each curriculum was correlated with the components of motivation, we used the Kendall tau correlation test. To determine the reliability of the questionnaire, we determined the alpha coefficient of Cronbach.

All statistical analyses were two-tailed and values of p less than 0.05 were considered statistically significant.

## Results

**Figure 1:**
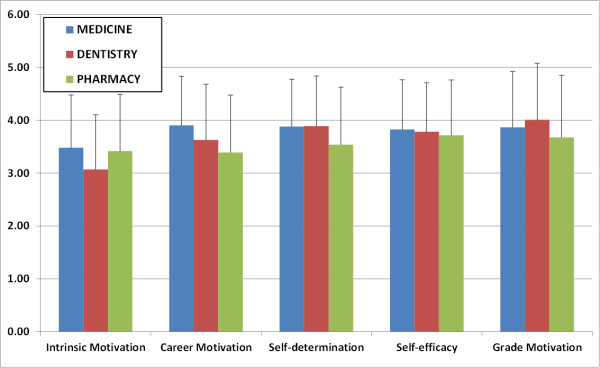
**Comparison of the results obtained for each component of motivation in each group of students.** Average values obtained for each component of motivation in the medicine, dentistry and pharmacy curricula are shown. Dispersion bars correspond to mean standard deviations.

In our results, the reliability of the scale was 0.8596 as determined by the Cronbach’s alpha coefficient. The average scores obtained for each component and for each item in each group of students (medicine, dentistry and pharmacy) are shown in Table 1, whereas the overall mean scores for each component of motivation are shown in Table 2. Figure 1 compares the data for each of the five components of motivation in each of the three groups of students according to degree program.

**Table 1:** Mean scores obtained for each item and each group of students

		**Medicine students**			**Dentistry students**			**Pharmacy students**		
		**All students**	**Female students**	**Male students**	**All students**	**Female students**	**Male students**	**All students**	**Female students**	**Male students**
**1. Intrinsic motivation**	**03. Learning histology is interesting**	3.85 ± 0.83	3.80 ± 0.91	3.95 ± 0.65	3.42 ± 0.92	3.38 ± 0.93	3.50 ± 0.90	3.74 ± 0.99	3.88 ± 0.95	3.39 ± 1.02
	**17. I am curious about discoveries in histology**	3.57 ± 0.96	3.58 ± 0.98	3.56 ± 0.93	3.04 ± 1.01	2.94 ± 1.03	3.23 ± 0.96	3.52 ± 1.05	3.69 ± 1.02	3.10 ± 1.01
	**01. The histology I learn is relevant to my life**	3.44 ± 0.84	3.34 ± 0.81	3.65 ± 0.87	3.29 ± 0.86	3.26 ± 0.89	3.35 ± 0.81	3.24 ± 1.02	3.37 ± 0.95	2.90 ± 1.14
	**12. Learning histology makes my life more meaningful**	2.68 ± 1.02	2.65 ± 1.09	2.74 ± 0.88	2.35 ± 1.03	2.43 ± 1.01	2.19 ± 1.05	2.88 ± 1.00	3.03 ± 0.97	2.52 ± 1.00
	**19. I enjoy learning histology**	3.86 ± 0.85	3.83 ± 0.91	3.93 ± 0.70	3.26 ± 0.99	3.22 ± 0.96	3.32 ± 1.05	3.71 ± 1.06	3.81 ± 1.04	3.47 ± 1.11
**2. Career motivation**	**07. Learning histology will help me get a good job**	3.15 ± 1.04	3.03 ± 1.03	3.40 ± 1.03	3.02 ± 1.11	3.10 ± 1.08	2.89 ± 1.17	2.91 ± 0.97	2.94 ± 0.98	2.84 ± 0.93
	**13. Understanding histology will benefit me in my career**	4.28 ± 0.75	4.21 ± 0.78	4.42 ± 0.70	3.90 ± 0.92	3.89 ± 0.94	3.91 ± 0.91	3.68 ± 1.12	3.79 ± 1.07	3.39 ± 1.20
	**10. Knowing histology will give me a career advantage**	4.23 ± 0.77	4.18 ± 0.72	4.33 ± 0.87	3.90 ± 0.91	3.85 ± 0.95	3.98 ± 0.85	3.64 ± 1.12	3.66 ± 1.08	3.58 ± 1.23
	**25. I will use histology problem-solving skills in my career**	3.79 ± 0.74	3.72 ± 0.77	3.93 ± 0.68	3.55 ± 1.04	3.51 ± 1.12	3.64 ± 0.89	3.29 ± 0.97	3.31 ± 0.98	3.23 ± 0.96
	**23. My career will involve histology**	4.08 ± 0.80	4.08 ± 0.76	4.09 ± 0.89	3.78 ± 1.02	3.75 ± 1.07	3.82 ± 0.95	3.44 ± 1.08	3.53 ± 1.10	3.23 ± 1.02
**3. Self-determination**	**22. I study hard to learn histology**	3.67 ± 0.86	3.73 ± 0.84	3.53 ± 0.91	3.71 ± 0.88	3.74 ± 0.89	3.66 ± 0.86	3.29 ± 0.98	3.40 ± 0.93	3.00 ± 1.05
	**16. I prepare well for histology tests and labs**	4.42 ± 0.67	4.47 ± 0.69	4.33 ± 0.61	4.54 ± 0.60	4.51 ± 0.61	4.61 ± 0.58	4.09 ± 1.17	4.25 ± 1.07	3.71 ± 1.35
	**05. I put enough effort into learning histology**	4.11 ± 0.87	4.15 ± 0.89	4.02 ± 0.83	4.05 ± 0.81	4.09 ± 0.85	3.98 ± 0.73	3.74 ± 0.99	3.86 ± 0.99	3.45 ± 0.93
	**11. I spend a lot of time learning histology**	3.55 ± 0.85	3.60 ± 0.82	3.47 ± 0.91	3.67 ± 0.93	3.67 ± 0.92	3.68 ± 0.96	3.17 ± 1.03	3.25 ± 1.04	2.97 ± 0.98
	**06. I use strategies to learn histology well**	3.67 ± 0.90	3.65 ± 0.94	3.70 ± 0.80	3.47 ± 1.07	3.40 ± 1.11	3.61 ± 0.97	3.39 ± 1.03	3.53 ± 0.98	3.06 ± 1.09
**4. Self-efficacy**	**18. I believe I can earn a grade of “A” in histology**	2.89 ± 1.06	2.81 ± 1.04	3.05 ± 1.09	3.07 ± 1.03	3.10 ± 1.02	3.02 ± 1.07	3.15 ± 1.07	3.12 ± 0.95	3.23 ± 1.33
	**14. I am confident I will do well on histology labs and projects**	4.23 ± 0.69	4.27 ± 0.70	4.14 ± 0.68	4.20 ± 0.76	4.10 ± 0.82	4.39 ± 0.62	3.92 ± 1.07	3.91 ± 0.99	3.94 ± 1.26
	**15. I believe I can master histology knowledge and skills**	3.74 ± 0.69	3.70 ± 0.66	3.83 ± 0.73	3.67 ± 0.68	3.60 ± 0.72	3.81 ± 0.59	3.75 ± 0.93	3.78 ± 0.85	3.68 ± 1.11
	**21. I am sure I can understand histology**	4.17 ± 0.69	4.10 ± 0.69	4.33 ± 0.68	3.91 ± 0.79	3.89 ± 0.81	3.95 ± 0.78	3.89 ± 0.96	3.91 ± 0.87	3.84 ± 1.16
	**09. I am confident I will do well on histology tests**	4.11 ± 0.79	4.09 ± 0.83	4.14 ± 0.71	4.07 ± 0.85	3.93 ± 0.89	4.34 ± 0.71	3.88 ± 1.01	3.78 ± 1.00	4.13 ± 1.02
**5. Grade motivation**	**04. Getting a good histology grade is important to me**	4.05 ± 0.86	4.18 ± 0.79	3.79 ± 0.94	4.25 ± 0.98	4.24 ± 1.00	4.27 ± 0.97	3.83 ± 1.11	3.92 ± 1.07	3.61 ± 1.20
	**08. It is important that I get an “A” in histology**	3.51 ± 1.31	3.62 ± 1.29	3.29 ± 1.35	4.02 ± 1.20	4.01 ± 1.19	4.02 ± 1.25	3.49 ± 1.25	3.49 ± 1.23	3.48 ± 1.31
	**20. I think about the grade I will get in histology**	3.51 ± 1.11	3.60 ± 1.07	3.33 ± 1.17	3.76 ± 1.09	3.64 ± 1.06	3.98 ± 1.11	3.56 ± 1.16	3.70 ± 1.16	3.20 ± 1.10
	**24. Scoring high on histology tests and labs matters to me**	4.27 ± 0.72	4.23 ± 0.72	4.35 ± 0.72	4.08 ± 0.88	4.05 ± 0.86	4.14 ± 0.90	3.83 ± 1.07	3.97 ± 1.01	3.48 ± 1.15
	**02. I like to do better than other students on histology tests**	4.00 ± 0.94	3.93 ± 0.96	4.14 ± 0.89	3.94 ± 1.12	3.96 ± 1.17	3.91 ± 1.03	3.66 ± 1.24	3.68 ± 1.23	3.61 ± 1.31

**Table 2:** Mean scores obtained for each component of motivation and each group of students

	**Medicine**			**Dentistry**			**Pharmacy**		
	**All students**	**Females**	**Males**	**All students**	**Females**	**Males**	**All students**	**Females**	**Males**
**1. Intrinsic motivation**	3.48 ± 1.00	3.44 ± 1.03	3.57 ± 0.92	3.07 ± 1.03	3.04 ± 1.02	3.12 ± 1.06	3.42 ± 1.07	3.55 ± 1.03	3.07 ± 1.10
**2. Career motivation**	3.91 ± 0.92	3.84 ± 0.93	4.03 ± 0.91	3.63 ± 1.05	3.62 ± 1.07	3.65 ± 1.03	3.39 ± 1.09	3.45 ± 1.08	3.25 ± 1.09
**3. Self-determination**	3.88 ± 0.89	3.92 ± 0.90	3.81 ± 0.87	3.89 ± 0.95	3.88 ± 0.97	3.91 ± 0.91	3.54 ± 1.09	3.65 ± 1.06	3.24 ± 1.11
**4. Self-efficacy**	3.83 ± 0.94	3.79 ± 0.95	3.90 ± 0.91	3.79 ± 0.92	3.72 ± 0.92	3.90 ± 0.91	3.72 ± 1.05	3.70 ± 0.98	3.77 ± 1.20
**5. Grade motivation**	3.87 ± 1.05	3.91 ± 1.02	3.78 ± 1.11	4.01 ± 1.07	3.98 ± 1.08	4.06 ± 1.06	3.68 ± 1.17	3.75 ± 1.15	3.48 ± 1.21

Statistical analysis showed that there were significant differences (p < 0.05) for the three comparisons between groups of students in the overall mean scores for components 2 and 5 (medicine vs. dentistry, medicine vs. pharmacy and dentistry vs. pharmacy students). In addition, we found significant differences in component 1 scores between dentistry and pharmacy students, and between dentistry and medical students. Significant differences (p < 0.05) were also found in component 3 scores between medical and pharmacy students, and between dentistry and pharmacy students (Table 3). Furthermore, significant differences were found for several specific items corresponding to all 5 components (Table 3).

**Table 3:** Statistical comparison of the scores assigned to each item and each factor by each group of students

		**Medicine vs. dentistry students**	**Medicine vs. pharmacy students**	**Dentistry vs. pharmacy students**	**Medicine vs. dentistry students**	**Medicine vs. pharmacy students**	**Dentistry vs. pharmacy students**
**1. Intrinsic motivation**	**03. Learning histology is interesting**	**0.0002**	0.5922	**0.0054**	**0.0000**	0.3681	**0.0000**
	**17. I am curious about discoveries in histology**	**0.0000**	0.7238	**0.0006**			
	**01. The histology I learn is relevant to my life**	0.1219	0.2172	0.8939			
	**12. Learning histology makes my life more meaningful**	**0.0080**	0.1246	**0.0001**			
	**19. I enjoy learning histology**	**0.0000**	0.3872	**0.0006**			
**2. Career motivation**	**07. Learning histology will help me get a good job**	0.4167	0.1062	0.4431	**0.0000**	**0.0000**	**0.0001**
	**13. Understanding histology will benefit me in my career**	**0.0008**	**0.0000**	0.1917			
	**10. Knowing histology will give me a career advantage**	**0.0033**	**0.0000**	0.0993			
	**25. I will use histology problem-solving skills in my career**	0.1091	**0.0001**	**0.0452**			
	**23. My career will involve histology**	**0.0253**	**0.0000**	**0.0194**			
**3. Self-determination**	**22. I study hard to learn histology**	0.5548	**0.0057**	**0.0011**	0.6183	**0.0000**	**0.0000**
	**16. I prepare well for histology tests and labs**	0.1503	0.1346	**0.0068**			
	**05. I put enough effort into learning histology**	0.4562	**0.0036**	**0.0155**			
	**11. I spend a lot of time learning histology**	0.2837	**0.0018**	**0.0002**			
	**06. I use strategies to learn histology well**	0.1792	**0.0495**	0.5744			
**4. Self-efficacy**	**18. I believe I can earn a grade of “A” in histology**	0.1567	**0.0423**	0.4716	0.3597	0.2132	0.6927
	**14. I am confident I will do well on histology labs and projects**	0.9061	0.0742	0.1023			
	**15. I believe I can master histology knowledge and skills**	0.7239	0.3638	0.1884			
	**21. I am sure I can understand histology**	**0.0095**	**0.0463**	0.6745			
	**09. I am confident I will do well on histology tests**	0.9163	0.1622	0.2041			
**5. Grade motivation**	**04. Getting a good histology grade is important to me**	**0.0151**	0.2364	**0.0015**	**0.0037**	**0.0093**	**0.0000**
	**08. It is important that I get an “A” in histology**	**0.0012**	0.8137	**0.0004**			
	**20. I think about the grade I will get in histology**	0.0706	0.6620	0.2132			
	**24. Scoring high on histology tests and labs matters to me**	0.1216	**0.0023**	0.1164			
	**02. I like to do better than other students on histology tests**	0.9226	0.0740	0.0870			

All values are p values with Mann-Whitney test. Statistically significant p values are highlighted in bold.

Moreover, significant differences (p < 0.05) between genders were found in medical students in overall mean scores for component 2, in dentistry students for component 4 and in pharmacy students for components 1, 3 and 5 (Table 4). In addition, significant differences (p < 0.05) were found for several specific items corresponding to components 1, 3, 4 and 5 (Table 4).

**Table 4:** Statistical comparison of the scores assigned to each item and each factor by male and female students

		**Medicine students**	**Dentistry students**	**Pharmacy students**	**Medicine students**	**Dentistry students**	**Pharmacy students**
**1. Intrinsic motivation**	**03. Learning histology is interesting**	0.4606	0.5850	**0.0153**	0.1267	0.2601	**0.0000**
	**17. I am curious about discoveries in histology**	0.8768	0.1280	**0.0054**			
	**01. The histology I learn is relevant to my life**	**0.0323**	0.4110	0.0625			
	**12. Learning histology makes my life more meaningful**	0.5722	0.2112	**0.0241**			
	**19. I enjoy learning histology**	0.6279	0.4688	0.1212			
**2. Career motivation**	**07. Learning histology will help me get a good job**	0.0694	0.2695	0.7512	**0.0108**	0.9104	0.0963
	**13. Understanding histology will benefit me in my career**	0.1537	0.9128	0.1217			
	**10. Knowing histology will give me a career advantage**	0.1168	0.5898	0.8420			
	**25. I will use histology problem-solving skills in my career**	0.1547	0.7221	0.7771			
	**23. My career will involve histology**	0.7377	0.9269	0.2347			
**3. Self-determination**	**22. I study hard to learn histology**	0.2119	0.4284	0.0859	0.1052	0.9748	**0.0001**
	**16. I prepare well for histology tests and labs**	0.1131	0.3225	**0.0290**			
	**05. I put enough effort into learning histology**	0.3118	0.2507	**0.0214**			
	**11. I spend a lot of time learning histology**	0.4152	0.9346	0.1913			
	**06. I use strategies to learn histology well**	0.7517	0.3795	**0.0489**			
**4. Self-efficacy**	**18. I believe I can earn a grade of “A” in histology**	0.3854	0.6038	0.6002	0.2053	**0.0130**	0.0929
	**14. I am confident I will do well on histology labs and projects**	0.2566	0.0692	0.4412			
	**15. I believe I can master histology knowledge and skills**	0.2669	0.1703	0.9704			
	**21. I am sure I can understand histology**	0.0770	0.6827	0.8237			
	**09. I am confident I will do well on histology tests**	0.8523	**0.0098**	**0.0436**			
**5. Grade motivation**	**04. Getting a good histology grade is important to me**	**0.0204**	0.8090	0.2013	0.1819	0.2887	**0.0151**
	**08. It is important that I get an “A” in histology**	0.1783	0.7497	0.9809			
	**20. I think about the grade I will get in histology**	0.1818	0.0775	**0.0289**			
	**24. Scoring high on histology tests and labs matters to me**	0.3413	0.4998	**0.0387**			
	**02. I like to do better than other students on histology tests**	0.2154	0.6028	0.8338			

All values are p values with Mann-Whitney test. Statistically significant p values are highlighted in bold.

When the overall mean scores for the different components were correlated with the final performance in histology of the students corresponding to the three curricula, we found a significant positive correlation (p < 0.05 and r = 0.666) for the components 2 and 4. Specifically, all 5 items in component 2 were positively correlated (p < 0.05 and r = 0.6666) with performance, whereas items 2, 4 and 5 were correlated with performance in component 4.

## Discussion

Evaluating the main components of motivation for learning histology in medical, dentistry and pharmacy students is important because it can help instructors to help their students, aid in monitoring their motivation to learn science, and support efforts to better organize collaborative learning activities based on an appropriate selection of highly motivated students [38]. In this connection it is worth noting that the elements essential for stimulating motivation in health science students appear to be absent as a primary aim in many curricular plans [1]. In the present work, we have used the SMQII questionnaire developed by Glynn and cols., which has been previously validated by these authors as one of the most accurate instruments for assessing motivation and its components, and showed high reliability as determined by the alpha coefficient of Cronbach obtained in our study.

One of the most innovative aspects of the SMQII is the transformation of the classical scale termed extrinsic motivation (or “learning science as a means to a tangible end”) into two scales, i.e. grade motivation and career motivation, which more clearly target the objectives that students perceive to be important in this stage of their education. This is especially important in health science education because the motivation to learn health science disciplines is influenced differently by short-term goals such as obtaining a high course grade and long-term goals such as success in their professional career practice, which in health science professions is explicitly regulated by professional and government bodies.

The results of the present study show that in relation specifically to learning histology, the profiles for components of motivation defined as career motivation and self-efficacy were similar in all three degree programs. The scores for both components were highest in the group of medical students, followed in decreasing order by dentistry and pharmacy. In the career motivation component there were significant differences when the overall mean scores for the three groups were compared (medicine vs. dentistry, medicine vs. pharmacy and dentistry vs. pharmacy students) whereas differences were not significant in self-efficacy. Interestingly, both profiles were positively correlated with the students’ final performance in histology, pointing out that the extent of career motivation and self-efficacy are clearly influencing the final outcome of the students in histology. Our study confirms some previous reports using different methods suggesting that self-efficacy is associated with effort, persistence, and performance [49,50]. The relationship between these two components of motivation and their correlations with each of the three groups of students reflects the relationship between the primary long-term goals that students focus on, which is what characterizes career motivation, and students’ beliefs about their capabilities in a specific area (e.g. histology), which is what characterizes self-efficacy and influences the choice of activities that allow individuals to decide which tasks to focus on [25,51-54].

In the health professions the long-term goal of professional career practice is an important component of motivation, which is related more closely with future competencies to be used in regulated professional practice than with knowledge and skills to be acquired in the learning process during university study. In this regard our results are consistent with several studies which found that compared to dentistry students, medical students had a more professional attitude, whereas dentistry students showed a greater commitment to personal and financial gain [29,55,56]. Regardless of these observations, the higher scores in our group of medical students are most likely related with a more highly developed system of regulations for professional practice. The lower scores among pharmacy students are most likely related with the fact that this area, according to Figgs and Cox [33] is a profession that lends itself to many career avenues. The intermediate scores in our group of dentistry students are most likely related with the lack of a sense of public service among these students –a perception that results in attitudes detrimental to the public perception of the dental profession as a whole [29]. The results we obtained with the SMQII for histology learning are consistent with our earlier finding of a high level of motivation for professional practice among medical students who were offered a choice of different learning methods [42].

In the present study the self-determination and grade motivation components yielded a similar motivational profile in all three groups of students. Mean scores for these components were highest in dentistry, followed in decreasing order by medicine and pharmacy. The differences between degree programs were statistically significant for the grade motivation, and between pharmacy and medicine students and between pharmacy and dentistry students for self-determination. The similarities in the response profiles for these components among the groups shows that a relationship exists between self-determination, and hence the control that students believe they have over their learning process, and the grade motivation, that is, the short-term primary goals of students enrolled in different degree programs. It is therefore unsurprising that dentistry students scored highest in both of these components of motivation, especially in grade motivation. Our results suggest, as Boiche and cols. [57] showed for physical education, an adaptive role for the self-determination component of motivation towards histology among dentistry and medicine students, with high levels of self-regulation thanks to which individuals do not act without feelings of control or competence to achieve success during their undergraduate degree program. Self-determination, i.e. the option to control the learning process for histology and therefore to self-regulate this activity during undergraduate education, is seen by dentistry and medicine students as a more attainable goal under these circumstances. When the overall mean scores for these two components were correlated with the final performance in histology of the students corresponding to the three curricula, we did not found a significant correlation. This could mean that self-determination and grade motivation will not determine the final score of the students enrolled in the histology matters.

The use of this questionnaire allowed us to analyze independently the two major components of the extrinsic motivation -career motivation and grade motivation-. Our data illustrates clear differences in the results obtained for these components among groups of students, and suggests that it may be possible to identify relationships between components that might otherwise be overlooked. In addition, our results show that both components have different influence on motivation for learning histology in health sciences curricula. Whereas the career motivation, which showed the same profile that self-efficacy, was correlated with the final outcome of the students, grade motivation, whose profile was similar to that of self-determination, was not correlated with the final performance.

Finally, intrinsic motivation showed the highest mean scores in medicine, followed in decreasing order by pharmacy and dentistry. The differences between medical and pharmacy students vs. dentistry students were statistically significant. The lower mean scores for this component in dentistry students vs. medicine and pharmacy students is likely related with the general perception, supported by several studies, that there is no statistical correlation between the amount of undergraduate basic science education and performance in dentistry school or on board exams, although dentistry graduates and practitioners do perceive the importance of these science a posteriori, including histology [34,58]. No correlation with the final histological performance was found for this component, thus confirming that this important component of motivation is not as relevant as career motivation and self-efficacy for the academic achievement.

Regarding the differences between genders, our results suggest that only one of the components of motivation was different in the case of medicine and dentistry students, although three components were statistically different in pharmacy students. Although Hulsman and cols. [44] reported no differences between males and females in the strength of motivation, more recent work by Kusurkar and cols. [46], showed that strength of motivation appears to be a dynamic entity, changing primarily with age and maturity and to a lesser extent with gender and experience. In the present study we found no differences between male and female medical students for any of the components except career motivation, which was higher in male than in female students, although none of the specific items in this component was significantly different between males and females. Among dentistry students there were no gender differences for intrinsic motivation, career motivation, self-determination and grade motivation, whereas men and women differed with regard to self-efficacy. Among pharmacy students we found gender differences for intrinsic motivation, self-determination and grade motivation but not for self-efficacy or career motivation. Although the differences between genders in the motivation to learn sciences remain poorly understood, they may result from factors such as role modeling and socialization by parents, teachers, peers and the media, and not from “innate or natural differences” between women and men [15,16,45].

Our findings with regard to the relative influence of different components of motivation in different groups of health science undergraduates are potentially useful in future efforts to answer some interesting questions. For example, are there gender differences in how students self-regulate in order to control their motivation and behavior? What role do the different components of motivation play in the overall motivation of males and females to perform well in these degree programs?

The motivational process is a substantially undervalued factor in curriculum development [1]. Because the motivation to learn, as conceptualized in social cognitive theory, is a multicomponent construct, determining the role of each component of motivation is useful not only to help individual students succeed in the learning process according to their motivational background, but also to help instructors develop appropriate teaching strategies based on their knowledge of specific motivational profiles among students enrolled in different programs.

Limitations of the present study are related to the lack of knowledge on the previous motivation of the students before being incorporated to the study, on their previous knowledge on histology and other related sciences that could influence their perceptions. In addition, we do not know to what extent we can generalize our findings to other students and other disciplines.

A strength of our study is that the histology course of interest was taught with the same methods by instructors from the same department to all participants, who were enrolled in different undergraduate programs at the same university. We feel our results contribute to our knowledge about the close relationships among different components of motivation in specific disciplines within different health sciences curricula. Ultimately, these findings can support improved efforts to develop more effective medical education curricula.

## Conclusions

Our results show that our histology students enrolled in each of three undergraduate health science programs differed in their personal motivational profiles. This finding is potentially useful to foster their learning process, because if they are metacognitively aware of their motivation they will be better equipped to self-regulate their science-learning behavior in histology. But our results also show that the overall motivational profile for learning histology differs among medical, dentistry and pharmacy students, and that instructors as well as education policy makers can use their awareness of these differences to enhance curricula so that they focus not only on fostering the cognitive component of learning but also integrate students’ levels and types of motivation into the processes of planning, delivery and evaluation of medical education. Although the differences between genders were not significant for many of the components of motivation, certain differences stand out. These differences suggest a need to increase our knowledge of possible variations and take advantage of them in developing both individually-targeted and curriculum-based teaching processes.

## Competing interests

The authors declare that they have no competing interests.

## Authors’ contributions

ACS participated in the design of the work, analysis of the results, discussion and writing the manuscript. JALN contributed to the study design, selection of literature and critical review of the manuscript. VC took part in the analysis and interpretation of the data, and literature review. MAMP participated in data collection and management of the results. TS contributed to writing the manuscript and critical review of the literature. MA contributed to design the work and interpret the data, revising the article and critically appraising the content. All authors have approved the final version of the article submitted.

## Authors’ information

ACS is a Master Degree and PhD student at the University of Granada. He is involved in education research with a focus on self-learning methodologies.

JALN is a professor in the Department of Didactics and School Organization at the Faculty of Education Sciences, University of Granada.

VD is a Master Degree and PhD in Tissue Engineering and a research fellow in the Department of Histology at the University of Granada, Pharmacy School.

MAMP is a Master Degree and PhD student at the University of Granada. He is involved in education research in Dentistry Schools.

TS is a full professor in the Department of Didactics and School Organization at the Faculty of Education Sciences, University of Granada.

MA is a full professor in the Department of Histology at the Medical School, University of Granada.

## Pre-publication history

The pre-publication history for this paper can be accessed here:

http://www.biomedcentral.com/1472-6920/14/46/prepub
